# Process optimization for green synthesis of silver nanoparticles using *Rubus discolor* leaves extract and its biological activities against multi-drug resistant bacteria and cancer cells

**DOI:** 10.1038/s41598-024-54702-9

**Published:** 2024-02-19

**Authors:** Saeed Ghasemi, Sara Dabirian, Faezeh Kariminejad, Diba Eghbali Koohi, Mehran Nemattalab, Sina Majidimoghadam, Ehsan Zamani, Fatemeh Yousefbeyk

**Affiliations:** 1 Department of Medicinal Chemistry, School of Pharmacy, Guilan University of Medical Sciences, Rasht, Iran; 2 Department of Pharmaceutical Biotechnology, School of Pharmacy, Guilan University of Medical Sciences, Rasht, Iran; 3 Department of Pharmacognosy, School of Pharmacy, Guilan University of Medical Sciences, Rasht, Iran; 4 Department of Pharmaceutics, School of Pharmacy, Guilan University of Medical Sciences, Rasht, Iran; 5 Department of Pharmacology and Toxicology, School of Pharmacy, Guilan University of Medical Sciences, Rasht, Iran

**Keywords:** Himalayan blackberry, Total phenols, Flavonoids, Multidrug-resistant bacteria, Cancerous cell lines, Drug screening, Cancer, Infectious diseases

## Abstract

Multi-drug resistant (MDR) bacteria are considered a serious public health threat. Also, increasing rate of resistance to anticancer drugs, as well as their toxicity, is another point of concern. Therefore, the new antibacterial and anticancer agents are always needed. The synthesizing silver nanoparticles (AgNPs) using medicinal plants, is an effective approach for developing novel antibacterial and anticancer agents. *Rubus discolor*, a native species of the Caucasus region, produces leaves that are typically discarded as a by-product of raspberry production. The present study has focused on optimizing the green synthesis of AgNPs using *R. discolor* leaves extract through response surface methodology. The optimal values for AgNPs synthesis were an AgNO_3_ concentration of 7.11 mM, a time of 17.83 h, a temperature of 56.51 °C, and an extract percentage of 29.22. The production of AgNPs was confirmed using UV–visible spectroscopy (λ_max_ at 456.01 nm). TEM analysis revealed well-dispersed AgNPs (an average size of 37 nm). The XRD analysis confirmed the crystalline structure. The EDX detected a strong peak at 3 keV corresponded to Ag. The zeta potential value (− 44.2 mV) indicated the stability of nanoparticles. FT-IR spectra showed the presence of various functional groups from plant compounds, which play an important role in the capping and bio-reduction processes. The AgNPs revealed impressive antibacterial activities against MDR *Escherichia coli* and *Pseudomonas aeruginosa* (MIC ranging from 0.93 to 3.75 mg ml^−1^). The phytochemical analysis indicated the presence of phenolics, tannins, and flavonoids on the surface of AgNPs. They also showed significant cytotoxic effects on A431, MCF-7, and HepG2 cells (IC_50_ values ranging from 11 to 49.1 µg ml^−l^).

## Introduction

The study and application of nanostructured materials, with sizes ranging from 1 to 100 nm, have been highlighted recently because they are widely applicable in many multidisciplinary fields^1–5^. Compared to bulk materials, nanoparticles (NPs) have a larger surface area to volume ratio and ultra-small size, which give them unique thermal, physiochemical, and biological properties^6,7^. These characteristics make them attractive candidates for developing countless applications in agriculture, biology, chemical engineering, biomedicine, and pharmaceutical industry^8^. According to their shapes, sizes, and properties, NPs have been classified into several groups, including carbon-based nanoparticles, polymeric nanoparticles, ceramic nanoparticles, and metal nanoparticles^9^. Among them, metal nanoparticles comprise gold, silver, copper, magnetic (cobalt, iron, and nickel), and semiconducting materials^2,10^. Silver nanoparticles (AgNPs) have gained significant attention based on various biological activities, including, antibacterial, antifungal, antiviral, anticancer, anti-inflammatory, and wound healing properties^11,12^. They have been used in food processing, cosmetics, home cleaning, catalytic and garment production, and pharmaceutical industry^13^.

There are various mechanical and chemical methods for producing nanomaterial that have several disadvantages, such as high cost, low yield, and being relatively complicated. Also, they are not environmentally friendly due to the application of toxic chemicals and solvents, and the production of dangerous by-products^6,9,14^. However, the green approaches for NPs production are eco-friendly, easy to apply, and safe for the environment, human beings, and living organisms^11,15^. Therefore, there is an increasing demand for developing safe methods to produce nanomaterials, using fungi, bacteria, or plants^6,16–19^. The plant extracts are preferred over other natural materials. Many natural compounds have been discovered in medicinal plants that play a crucial role in the preparation of nanoparticles^12^. The plant’s secondary metabolites, such as phenolic compounds, tannins, flavonoids, anthraquinones, carbohydrates, alkaloids, alkynes, allylic benzenes, ascorbic acids, alcoholic compounds, sugars, amides, amino acid residues, carotenes, steroids, saponins, and triterpenoids have proven to be able to reduce silver nitrate to AgNPs^7,12,20^. Recent investigations have revealed that NPs synthesized by bioactive phytochemicals possess more beneficial and effective properties than traditional herbal drugs^12^.

The World Health Organization (WHO) has recently declared antibacterial resistance as one of the three main risks to human health^21^. Drug-resistant pathogens kill about 700,000 people worldwide each year, and this number could increase to 10 million deaths a year by 2050^22^. Presently, the rapid development of drug-resistant strains of microorganisms as well as the serious lack of effective antibiotics, have revealed that the discovery and development of novel antimicrobial agents are logically necessary^22,23^. Silver is an inorganic antibacterial agent, which is nontoxic and safe. It is effective against 650 types of pathogenic microorganisms^24^. Studies showed that AgNPs can exert significant antibacterial activity against MDR (multi-drug resistant) bacteria by several mechanisms, such as inhibition of the cell respiration chain, disrupting the cellular signal transduction pathways, and generating reactive oxygen species (ROS), which causes toxicity in cells^12,25–27^.

Moreover, cancer is the second leading cause of death universally after cardiovascular diseases^28^. The American Cancer Society predicted that the worldwide burden of cancer will surge to 21.7 million fresh cases by the year 2030^29^. Today, there is growing attention to discovering inexpensive and more cost-effective drugs using natural resources like medicinal plants. The plants have been provided various new approaches for the cancer treatment, including green synthesis of AgNPs^29^.

Blackberries, *Rubus* spp., Rosaceae family, are widely distributed and cultivated worldwide and are of growing commercial relevance^30^. This genus comprises over 750 species in the world. The sweet taste fruits of many species are popular as a healthy and nutritious food, containing various phenolic compounds, dietary fiber, vitamin C, α-tocopherol, and carotenoids^31,32^. Also, blackberry leaves have been used traditionally, in form of tea or as a mouthwash, and gargle solution. It is reported that blackberry leaves have important bioactive components, such as phenols, flavonoids, tannins, terpenoids, and other anti-aging and antioxidant compounds, and can serve as a potential source for use in the food, pharmaceutical industry, and cosmetic^32,33^. In the flora of Iran, eight species of blackberry have been reported^34^. *Rubus discolor* Weihe & Nees., commonly known as Himalayan blackberry, is a native species of the Caucasus region of Eurasia^35^. It is widely distributed in the North and Northwest of Iran as a common weed^36^. Since the *Rubus* leaves are significantly consumed less than fruits, a large number of leaves are disposed of as a by-product of raspberry production^32^.Currently, the assessment of bioactive phytochemicals in the eliminated plant material has attracted great interest, because these by-products have high levels of constituents with biological properties^37^. As a result, the ecofriendly synthesis of AgNPs from the leaves which have potential biological activities could be of interest.

In the present study, the aqueous extract of *Rubus discolor* leaves was used for the biosynthesis of the AgNPs. The synthesis condition was optimized by response surface methodology (RSM). The nanoparticles were characterized by UV–Vis and FT-IR spectroscopic methods, as well as XRD, DLS, SEM–EDX, and TEM methods. The preliminary phytochemical investigations and the determination of total phenolic, tannin, and flavonoid contents were performed. The antibacterial activity was tested against two ATCC Gram-positive (*Streptococcus aureus* and *Bacillus subtilis*) and two ATCC Gram-negative bacteria (*Escherichia coli* and *Pseudomonas aeruginosa*). Also, ten MDR isolates of *E. coli* and *P. aeruginosa* were tested to investigate their susceptibility to the synthesized AgNPs. The cytotoxic activities of AgNPs and extract were investigated against three cancerous cell lines, including MCF-7 (breast cancer), A431 (epidermoid carcinoma), and HepG2 (liver hepatocellular carcinoma) as well as a normal cell line (HU02) by MTT assay.

## Results and discussion

### Phytochemical investigation of the aqueous extract and AgNPs

The preliminary phytochemical analysis of the aqueous extract of leaves revealed the presence of flavonoids, tannins, steroids, and carbohydrates (Table 1).

**Table 1:** Qualitative analysis of phytochemicals in *R. discolor* leaves extract.

Phytochemicals	Result
Flavonoids	+
Tannins	+
Steroids	+
Carbohydrates	+
Alkaloids	−
Coumarins	−
Quinone	−

+: presence, −: absence.

The total phenolic content (TPC) of the aqueous extract and AgNPs were calculated based on the gallic acid standard curve equation (y = 0.000899x − 0.0355, R^2^ = 0.999), using the Folin-Ciocalteu method. Also, the total flavonoid contents (TFC) were measured, based on the quercetin standard curve (y = 0.0192x − 0.0198, R^2^ = 0.995). The total tannin contents (TTC) were measured using the following standard curve plotted for tannic acid (as standard compound): y = 0.005x + 0.0281; R^2^ = 0.995. All the results are depicted in Table 2. According to the results, AgNPs showed lower TPC, TTC, and TFC than the aqueous leave extract. The reduction in polyphenolic contents was also reported in other studies^7,38,39^. The suggested reason is that the phenolic, tannin, and flavonoid compounds in the extract were consumed in the reduction process in the green synthesis of the AgNPs. Moreover, the concentration of these secondary metabolites determines the kinetics of the reaction, shape, and size of AgNPs^38^.

**Table 2:** Total phenolic content (TPC), total flavonoid content (TFC), and total tannin content (TTC) of AgNPs and aqueous extract of *Rubus discolor.*

Sample	TPC*	TFC**	TTC***
extract	57.31 ± 0.02*	27.63 ± 0.11**	114.12 ± 0.21***
AgNPs	35.54 ± 0.22	15.81 ± 0.09	73.2 ± 0.51

*mg GAE/g extract; **mg QE/g extract; ***mg TAE/g extracts.

### Statistical process optimization of green synthesis AgNPs using RSM

The results of the central composite design (CCD) for optimizing Ag synthesis conditions, including AgNO_3_ concentration, time, temperature, and the extract percent, were represented in Table 3.

**Table 3:** Coded values of used independent variables in response surface central composite design matrix and the observed response (absorbance) for AgNPs.

Run	A	B	C	D	Absorbance (456 nm)
1	− 1	1	1	1	1.42
2	1	− 1	1	1	2.01
3	1	1	− 1	1	1.84
4	1	− 1	− 1	− 1	1.04
5	1	1	1	− 1	1.44
6	− 1	1	− 1	− 1	0.92
7	0	0	0	0	1.76
8	− 1	− 1	− 1	1	0.68
9	− 1	− 1	1	− 1	1.13
10	0	0	0	0	1.68
11	1	1	1	1	2.58
12	− 1	− 1	− 1	− 1	0.84
13	− 1	1	1	− 1	0.98
14	1	− 1	− 1	1	1.92
15	0	0	0	0	1.68
16	− 1	1	− 1	1	0.72
17	1	− 1	1	− 1	1.36
18	1	1	− 1	− 1	1.12
19	0	0	0	0	1.83
20	− 1	− 1	1	1	1.32
21	0	0	2	0	1.85
22	2	0	0	0	2.24
23	0	2	0	0	2.08
24	0	0	0	2	2.15
25	0	0	0	0	1.88
26	0	0	− 2	0	1.04
27	0	0	0	0	1.76
28	0	− 2	0	0	1.12
29	0	0	0	− 2	1.28
30	− 2	0	0	0	0.84

A set of 30 runs based on the formula 2^N^ + 2 N + X was conducted, where N is the number of selected factors with 2^N^ factorial (16 runs), 2N axial (8), and X center points repetitions (6 runs). The Eq. 1 shows the correlation between the absorbance at 456 nm (as an indicator of SPR) and the four studied parameters in coded terms:

1 $$Y=1.77+0.34A+0.11B+0.2C+0.23D+0.036AB-0.014AC+0.19AD+0.03BC+0.034BD+0.074CD-0.097{A}^{2}-0.082{B}^{2}-0.12{C}^{2}-0.053{D}^{2}$$

where Y is the absorbance at 456 nm; A is AgNO_3_ concentration; B is the time of reaction; C is temperature; and D is the extract percentage. The analysis of variance (ANOVA) was carried out to investigate the suitability of the obtained model (Table 4).

**Table 4:** ANOVA analysis of CCD for the synthesized AgNPs.

Source	Sum of squares	*df*	Mean square	F Value	Prob > F	Significance
Block	0.38	2	0.19			
Model	8.68	14	0.62	30.34	< 0.0001	Significant
A-Ag Conc	3.98	1	3.98	194.65	< 0.0001	Significant
B-Time	0.51	1	0.51	25.12	0.0002	Significant
C-Temp	1.09	1	1.09	53.25	< 0.0001	Significant
D-Extract Percent	1.67	1	1.67	81.71	< 0.0001	Significant
AB	0.01	1	0.01	0.26	0.6205	
AC	0.02	1	0.02	0.93	0.3536	
AD	0.42	1	0.42	20.52	0.0006	Significant
BC	0.02	1	0.02	0.93	0.3536	
BD	0.01	1	0.01	0.51	0.4860	
CD	0.05	1	0.05	2.53	0.1355	
A^2^	0.44	1	0.44	21.50	0.0005	Significant
B^2^	0.21	1	0.21	10.06	0.0074	Significant
C^2^	0.43	1	0.43	21.08	0.0005	Significant
D^2^	0.19	1	0.19	9.21	0.0096	Significant
Residual	0.27	13	0.02			
Lack of Fit	0.24	10	0.02	3.38	0.1723	
Pure Error	0.02	3	0.01			
Cor Total	9.33	29				

Based on the statistics, a quadratic model was suggested to relate the experimental factors and their combinations and the response. The high F-value (30.34) and the low *p* value (*p* < 0.0001, only 0.01% chance of noise) showed that the obtained model is significant and acceptable. The variables A, B, C, D, AD, A^2^, B^2^, C^2^, and D^2^ were the significant parameters on the basis of _*p*_ value (p < 0.05). The F-value lack of fit was 3.38 with a *p* value of 0.1723 (chance of noise 17.23%), which shows the model is valid.

Three parameters, including the calculated determination coefficient (R^2^ and adjusted R^2^) and adequate precision, were used to evaluate the model’s efficacy of R^2^ and adjusted R^2^ values of 0.9703 and 0.9383, respectively, showed that the model has high efficacy and can properly explain the variability. Adequate precision (AP) of 19.305 (AP > 4 is desirable), which shows the signal-to -noise ratio, indicated adequate signal-to-noise. The Predicted R^2^ of 0.7957 showed a high correlation between predicted and observed responses. It should be in reasonable agreement with adjusted R^2^ (within the range of 0.2 adjusted R^2^).

**Figure 1:**
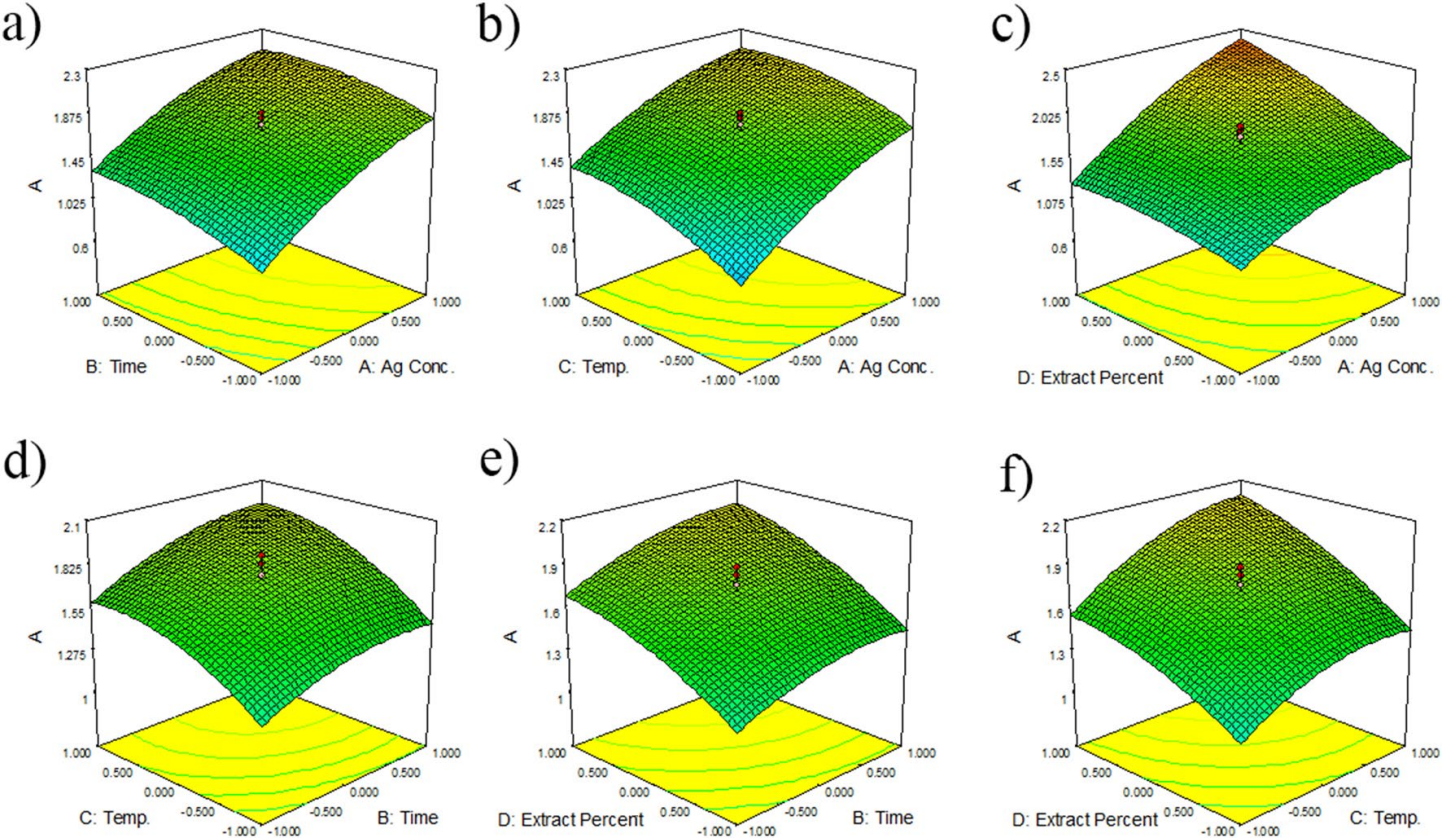
Three dimensions surface plots of AgNPs biosynthesis (**a**–**f**): Interaction effects of AgNO_3_ concentration (**A**), time (**B**), temperature (**C**), and extract percent (**D**) on the maximum absorbance value (Amax).

For evaluation of the best condition for each factor to obtain the maximum AgNPs yield, the 3D surface and contour plots were used (Fig. 1). These plots were on the basis of the corresponding interactions of two factors, while the third parameter was fixed at the optimum condition. The shape of the 3D contour plot shows the interaction significance.

Figure. 1a–c shows that AgNO_3_ concentration (A) had a significant effect on the AgNPs synthesis. When AgNO_3_ concentration increased, the yield of AgNPs increased depending on the second parameter. Othman et al. reported that AgNO_3_ concentration strongly affected the yield of AgNPs synthesis when interacting with other factors such as reaction pH value^40^. Likewise, El-Rafie showed that increasing the silver nitrate concentration dramatically increased the absorbance intensity^41^. Figures 1a, e, and f show that the time of the reaction (B) had a lesser momentous influence on yield of AgNPs synthesis in the interaction with AgNO_3_ concentration (A), temperature (C), and extract percent (D). When the reaction time increased, the AgNPs biosynthesis increased by the interaction of the second factor, including A, C, or D. Figures 1b, d, and f prove that temperature (C) had a stronger effect on the AgNPs biosynthesis than the time of reaction. Also, in a study, the AgNPs were synthesized using *Plantago major* extract, and it was showed that temperature had higher effect on the absorbance in comparison with time^42^. Figures 1c, e, and f explain that extract percent (D) had the second rank in the AgNPs synthesis after AgNO_3_ concentration. The yield of AgNPs biosynthesis increased with the increase in extract percent due to higher reducing agents in the reaction mixture^43^. A strong interaction was observed between A and D, and other interactions were not significant. The 3D surface plots showed that the effects of the four studied parameters on the AgNPs green synthesis were not equal, and the order of factors was as follows: A > D > C > B, respectively. In the optimized condition, the selected experimental model was tested using AgNO_3_ concentration of 7.11 mM, time of 17.83 h, temperature of 56.51, and extract percent of 29.22. The predicted absorbance at 456 nm was 1.92, which is close to the experimental value (2.12) that indicates the validity of the models. The yield of the reaction, in optimized condition was 53.31%.

## Characterization of AgNPs

### Optical properties and UV–Vis spectroscopy of the synthesized AgNPs

**Figure 2:**
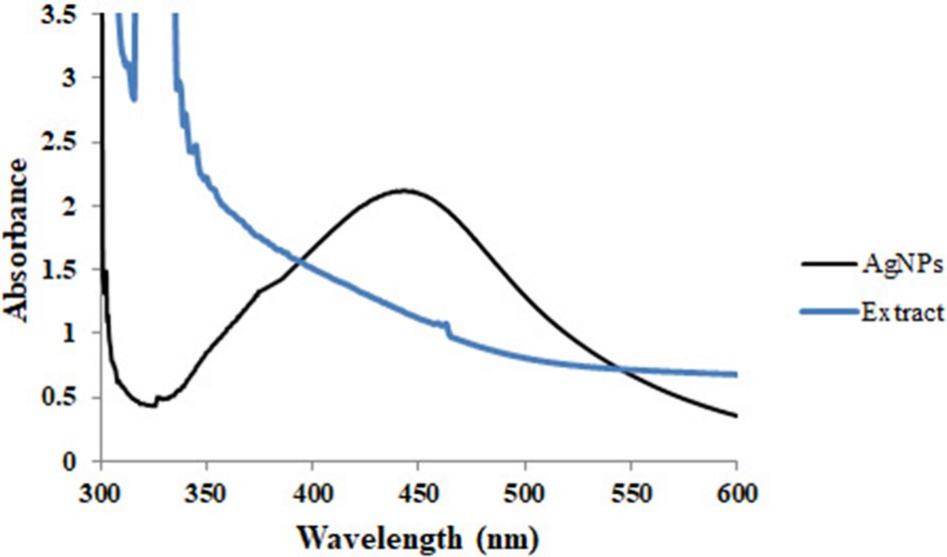
UV–Vis spectrum of the *R. discolor* extract and AgNPs.

The formation of AgNPs was first characterized by the observation of color change from pale yellow to dark brown, that revealed the Ag^+^ reduction into Ag^0^ nanoparticles. The color transformation is due to AgNPs’ optical properties and known as the localized surface plasmon resonance (SPR)^44^. Various factors like particle type, size, shape, morphology, dielectric environment, and composition have an impact on SPR. Also, UV–Vis spectroscopy is a common characterization tool to detect the SPR absorption peak of NPs and demonstrate their formation^45^. As depicted in Fig. 2, the UV–V is spectrum of AgNPs showed a SPR at 456.01 nm. In a study, Said et al. reported that the UV–vis spectrum of the AgNPs they produced was observed at 460 nm^16^. Additionally, Patra et al. revealed that their AgNPs had a maximum absorbance peak at 456 nm^46^. These findings are consistent with our study.

The numerous phytochemicals present in the aqueous extract of *R. discolor* could be responsible for the rapid bioreduction and capping of synthesized AgNPs. Typically, the bioactive compounds such as vitamins, flavonoids, tannins, phenolic acids, proteins, etc., are responsible for the fast reduction of Ag^+^, and control the size distribution and morphology of synthesized NPs^47^. According to the results of this study, the leaves extract contained tannins, flavonoids, steroids, and carbohydrates, which can act as reducing agents.

### TEM analysis

**Figure 3:**
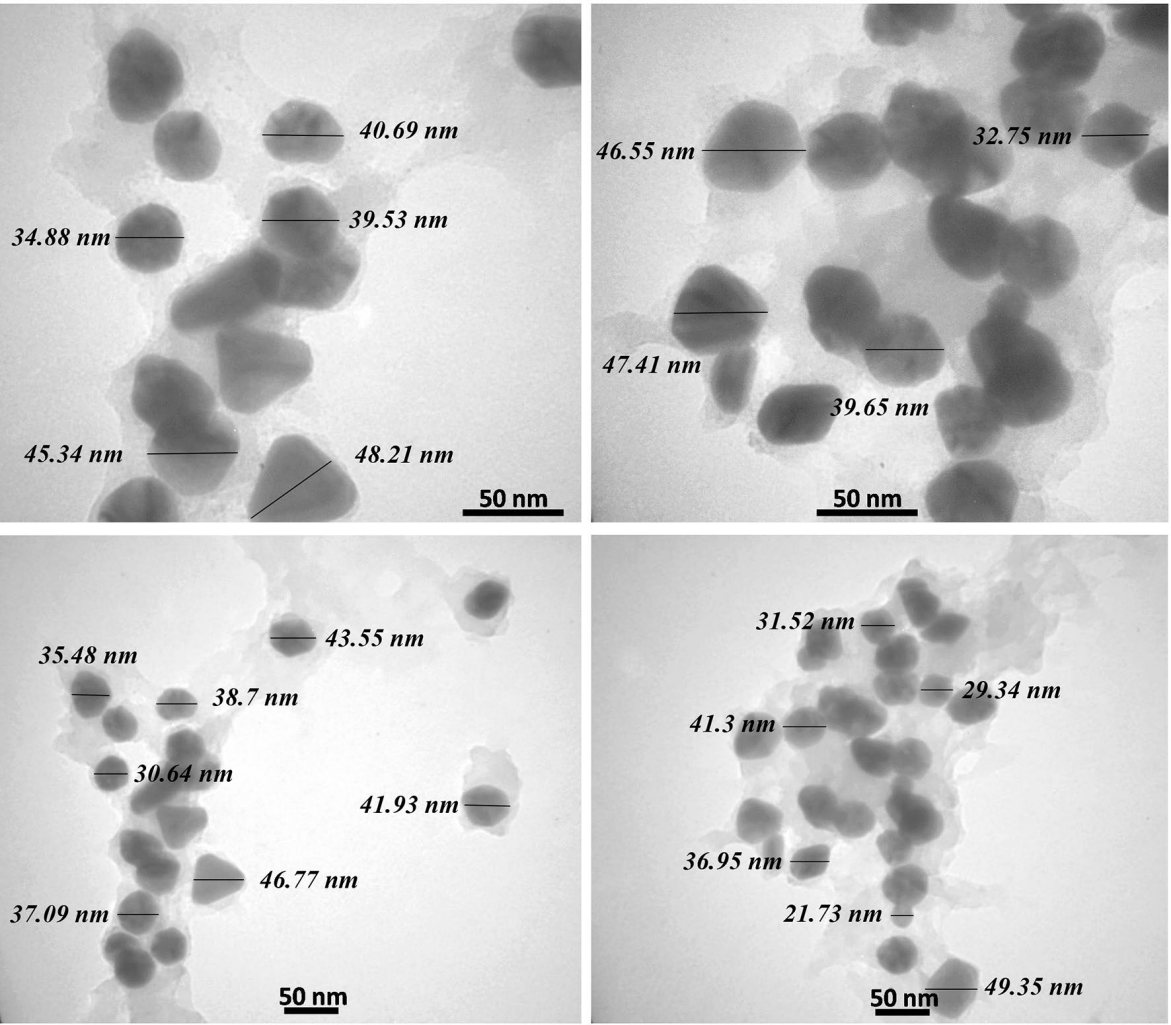
TEM images of AgNPs at optimized condition.

The AgNPs were evaluated by the transmission electron microscopes (TEM) for elucidation of the size, shape, and morphology. The microphotographs displayed that the nanoparticles were well-distributed and roughly spherical, with polydispersity, and without agglomeration. The size of the most particles ranged between 20 and 50 nm, with an average size of 37 nm (Fig. 3). It can be suggested that during the reaction, the content of reducing agent in plant extract deceased gradually, which led to the formation of AgNPs in different sizes. Also, careful observation of TEM images revealed no direct connection among AgNPs, even within the aggregates, presenting that AgNPs were surrounded by a thin layer of natural phytochemicals like amino acids and phenolic compounds^7,38^. In a study, Mariadoss et al. reported that the morphology of the AgNPs synthesized by the extract of *Malus domestica* was spherical, with polydispersity and a size ranging from 40 to 100 nm^48^. Also, Yassin et al. synthesized AgNPs from *Origanum majorana*, which showed polydispersity, with a size range from 10 to 60 nm^8^.

### SEM–EDX analysis

**Figure 4:**
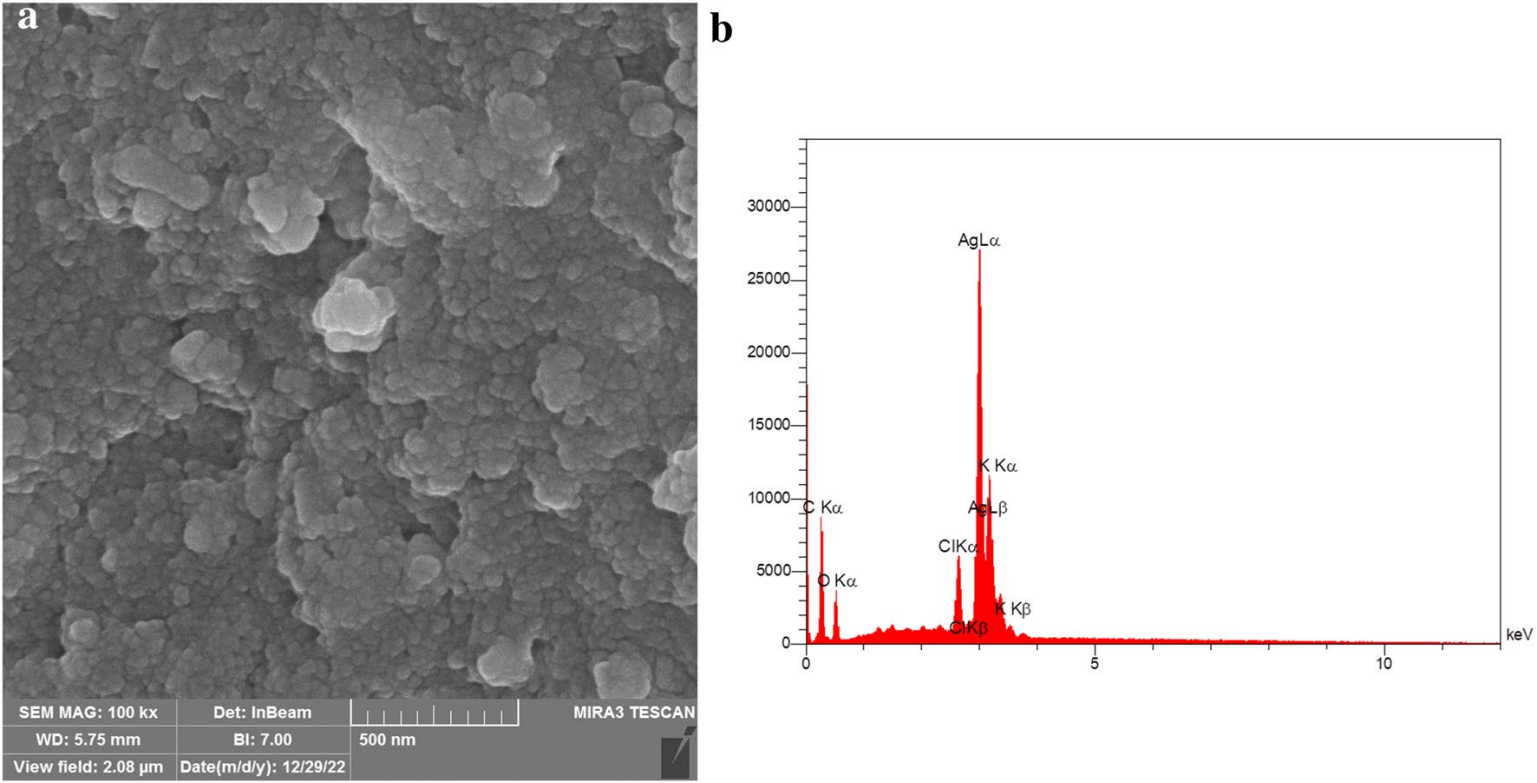
SEM image (**a**) and EDX spectrum (**b**) of biosynthesized AgNPs.

The surface nature and the elemental configuration of the AgNPs were determined by Scanning Electron Microscopy (SEM) with Energy Dispersive X-Ray (EDX) analysis. The SEM image displayed that AgNPs were polydisperse, about 38 nm in size, and predominantly spherical in shape (Fig. 4). The EDS analysis of the AgNPs was conducted to study the elemental composition of AgNPs. EDX analysis displayed a strong signal at 3.0 keV, which is characteristic of metallic Ag because of surface plasmon resonance, associated with the Ag-L_a_ line^49^. Additionally, the profile exhibited peaks for oxygen and carbon which could be attributed to the phytoconstituents attached to the surface of the AgNPs. Our results were in accordance with those of Okaiyeto et al., which produced AgNPs using aqueous extract of *Oedera genistifolia*, showing the presence of intense peak of silver element at 3.0 keV^50^. Moreover, Patra et al. synthesized AgNPs from *Pisum sativum*. The EDX spectra of their study showed the elemental composition of the AgNPs, with a strong peak at 3 keV that corresponded to Ag^46^.

### Zeta potential and DLS analysis

**Figure 5:**
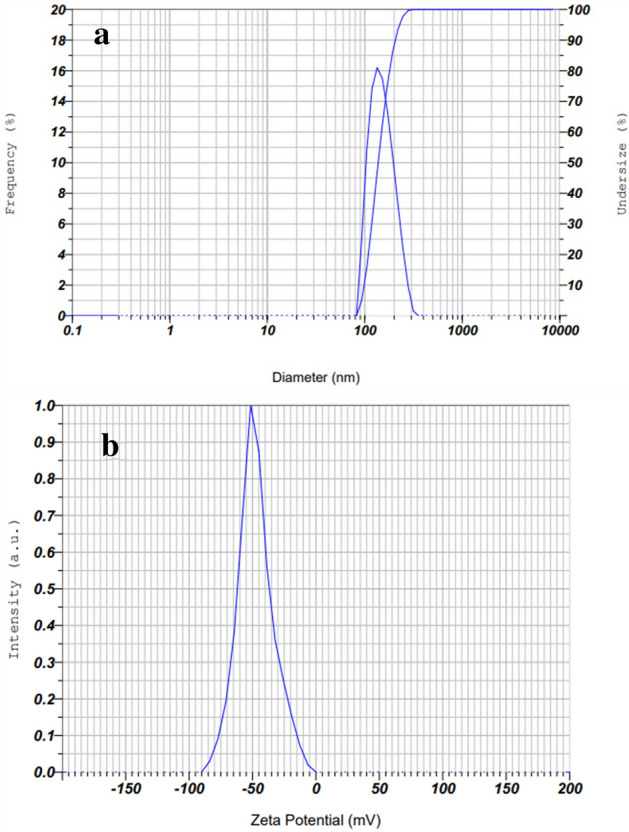
Size (**a**) and zeta potential value (**b**) of AgNPs prepared by *R. discolor.*

The particle size distribution of biosynthesized AgNPs was determined using a dynamic light scattering (DLS). The DLS determines the hydrodynamic size of colloids, and can estimate the average size of the nanoparticles in the mixture, approximately^51^. The results of this study indicated that the particle size and poly-dispersity index (PDI) values of AgNPs were 151.7 nm and 0.25, respectively (Fig. 5). As reported in previous studies, PDI (also known as heterogeneity index) shows the non-uniformity of particles in a colloidal solution. This value is unitless and is considered between 0.05 and 0.7. A PDI value close to 0.05 indicates that the particles are monodispersed, while colloidal solutions with PDI values close to 0.7 are heterogenous^51,52^. In this study, the reported PDI value of 0.25 is acceptable. On the other hand, the zeta potential (ζ) is one of the important factors for the characterization of the stability of nanoparticles in a solution. Nanoparticles with zeta potentials larger than + 30 mV and less than − 30 mV show considerable stability for colloidal dispersions^51^. The value of AgNPs zeta potential was − 44.2 mV. The highly negative value of ζ proved that the synthesized AgNPs had high stability^53^. Since, in this study, the external stabilizers were not used, meaning that the plant phytochemicals acted not only as the reducing agents of the Ag^+^ to Ag^0^, but also stabilized the synthesized nanoparticles.

It is obvious that the particle size in the DLS analysis is larger than in the TEM test. The DLS measurement is carried out in a fluid phase. This means that the AgNPs particles are in constant movement because of Brownian motion. Also, AgNPs have a charge on their surfaces, and consequently, they can interact with other ions, molecules, and surfaces, which contributes to the creation of adsorbed layers on the surface of the nanoparticles^14^. Therefore, the DLS shows the hydrodynamic diameter of the biomolecules surrounding AgNPs and the intensity-weighted average particle size^44^. However, the TEM image is taken in a dry state. Thus, the results of DLS and TEM cannot be in line with each other, and the results of DLS showed a normally larger size than those of TEM^14^.

### X‑Ray Diffraction Spectroscopy

**Figure 6:**
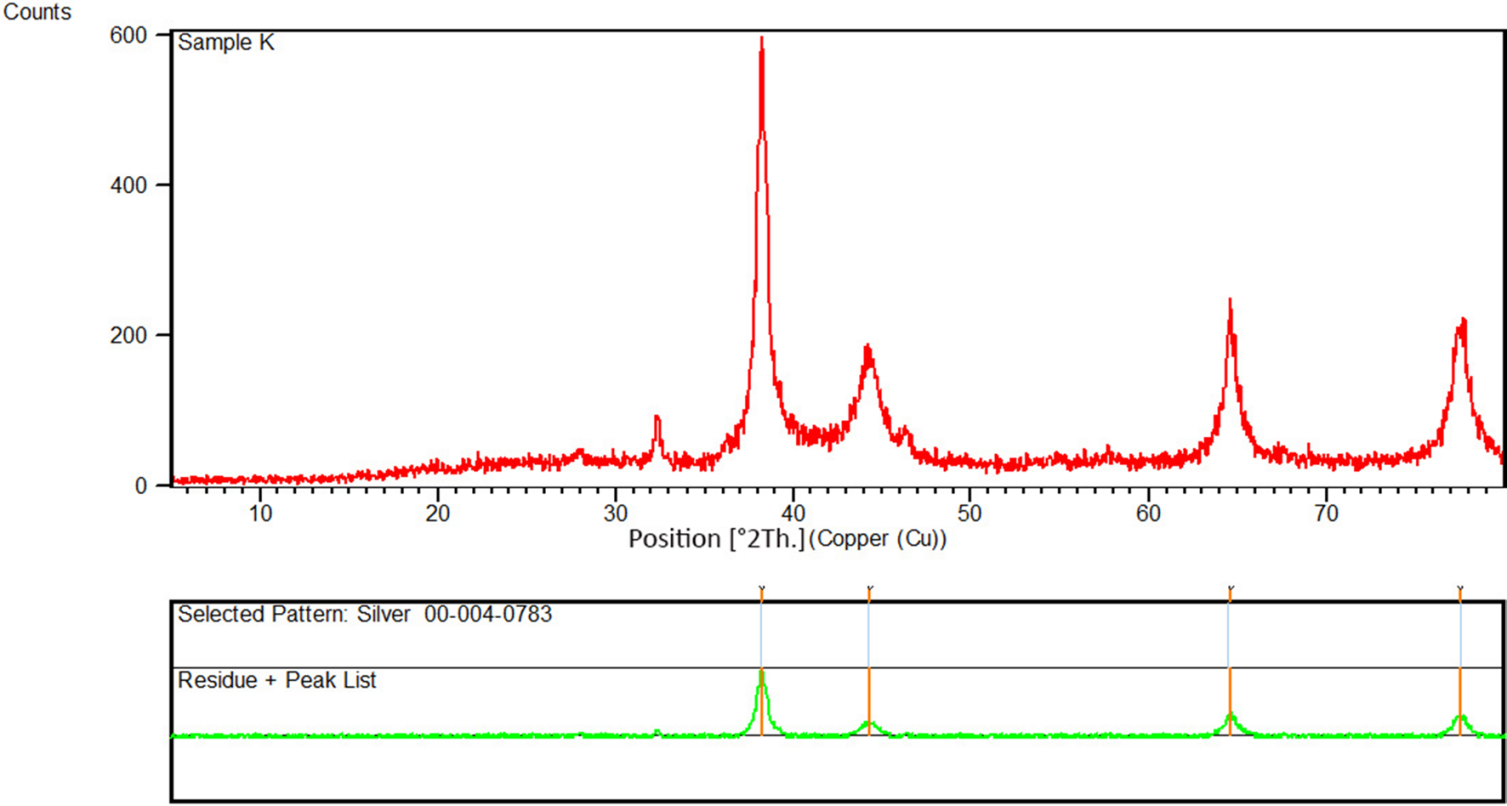
XRD pattern of green synthesized AgNPs.

The X-ray diffraction (XRD) pattern of AgNPs synthesized using aqueous extract of *R. discolor* is shown in Fig. 6. The peaks at 2 theta (θ) degrees of 38.1°, 44.2°, 64.5°, and 77.6° could be related to (111), (200), (220), and (311) facets, respectively, which corresponded to the database of the Joint Committee on Powder Diffraction Standards (JCPDS), file No. 00-004-0783. Debye–Scherrer formula (Eq. 2) was used to calculate the size of AgNPs, as follow:

2 $$D=\frac{0.94\lambda }{\beta COS\theta }$$

where D is the average crystallin size of AgNPs, λ is the wavelength of X-ray which is 0.1546 nm, β is the width at half maximum of the peak in radians, and θ is Braggs angle in degrees^54^.Similar to previous studies, it shows that synthesized AgNPs are face-centered cubic. Additional peaks on the XRD spectrum could be correlated to the crystallization of the plant phytochemicals coating the AgNPs^38,44^. The average size of AgNPs was 18 nm, which complied with the result from TEM images. These findings were consistent with previous study by Said et al. that reported the formation of AgNPs by detecting diffraction peaks at 2θ degrees of 38.1°, 44.2°, 64.4°, and 77.2°, which are corresponded to the planes (111), (200), (220), and (311), respectively. Similarly, Yassin et al. reported AgNPs, with the face-centered cubic structure and diffraction peaks at 2θ degrees of 38.18◦, 44.36◦, 64.35◦, and 77.54◦, which are related to reflection planes of (111), (200), (220), (311), respectively^8^.

### FT-IR analysis

**Figure 7:**
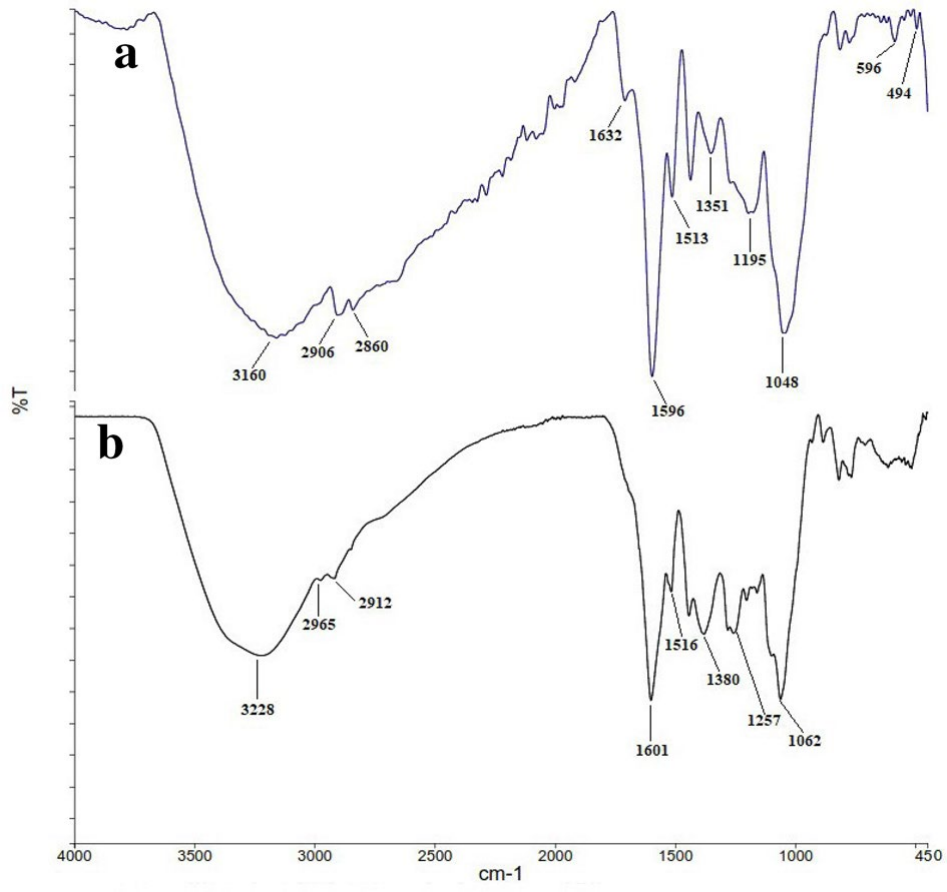
FTIR spectra of (**a**) AgNPs and (**b**) extract.

FT-IR analysis was performed to determine the structure of phytochemicals, exciting in aqueous leaves extract of *R. discolor*, which are responsible for surface coating and stabilization of the AgNPs. Figure 7 shows the IR spectra of the aqueous extract and the synthesized AgNPs.

The wavenumbers of different functional groups are summarized in Table 5. The characteristic peaks are determined by comparing peaks with the FT-IR results of other studies that have biosynthesized AgNPs using green methods.

**Table 5:** FT-IR analysis of *R. discolor* extract and the green synthesized AgNPs displaying different functional groups.

Absorption Peak (cm^−1^)		Assignment	References
AgNPs	Extract		
3160	3228	O–H and N–H stretching vibrations	Aref et al.^55^ Abdel-Raouf et al.^56^ Yousefbeyk et al.^7^ Salem S.^18^
2906 and 2860	2965 and 2912	asymmetric and symmetric stretching vibrations of aliphatic C–H	
1632	1601	C=O stretching vibration of amides	
1596	1601	bending vibration of amide I (CONH2), stretching vibration of C=C groups, and C–H bending vibration of alkenes	
1513	1516	out-of-plane bending vibration of N–H amide II	
1351	1380	out-of-plane bending vibration of N–H amide III	
1195 and 1048	1203 and 1062	overlapped stretching vibrations of C–O, C–N, C–O–C, and C–O–P	
< 1000	< 1000	Bending vibrations of sp2 C–H of alkenes and aromatic rings	

By comparison the FT-IR spectra of AgNPs and the leaves aqueous extract, it was demonstrated that some peaks were shifted. Also, the intensity of some peaks reduced or increased, and the appearance of several new peaks changed significantly. For example, peaks at 3228, 1601, 1516, and 1380 cm^−1^, corresponding to O–H and N–H stretching vibrations, shifted to 3160, 1596, 1513, and 3151 cm^−1^, respectively. That could be due to some electrostatic interactions among the AgNPs and functional groups of capping agents. Moreover, in the FT-IR spectrum of AgNPs, the appearance of a peak at 1632 cm^-1^, as well as increasing the intensity of the peak at 1436 cm^-1^, which are attributed to carbonyl vibrations, designated that the reduction of the silver ions is due to the oxidation of the hydroxyl groups to the carbonyl groups in the plant extract. The reduced peak intensity at 3160 cm^−1^ revealed the important role of OH and N–H in the reduction and binding mechanism^7^. Finally, new peaks at 596 and 494 cm^−1^ may be attributed to the bonding of AgNPs with phytochemicals in the extract. Similar to our study, Said et al. reported peaks at 775 to 540 cm^−1^ which were correspondent to the bonding of AgNPs to functional groups in the extract^16^. Also, Aref et al. suggested that the peaks at 488 and 407 cm^−1^ may refer to the binding of AgNPs to phytochemical groups^55^. Moreover, Yassin et al. showed that the AgNPs synthesized by *Origanum majorana* had characteristic peaks for functional groups such as phenolic, amines, hydroxyl, and alkyl groups.

### Suggested mechanism of formation of AgNPs

**Figure 8:**
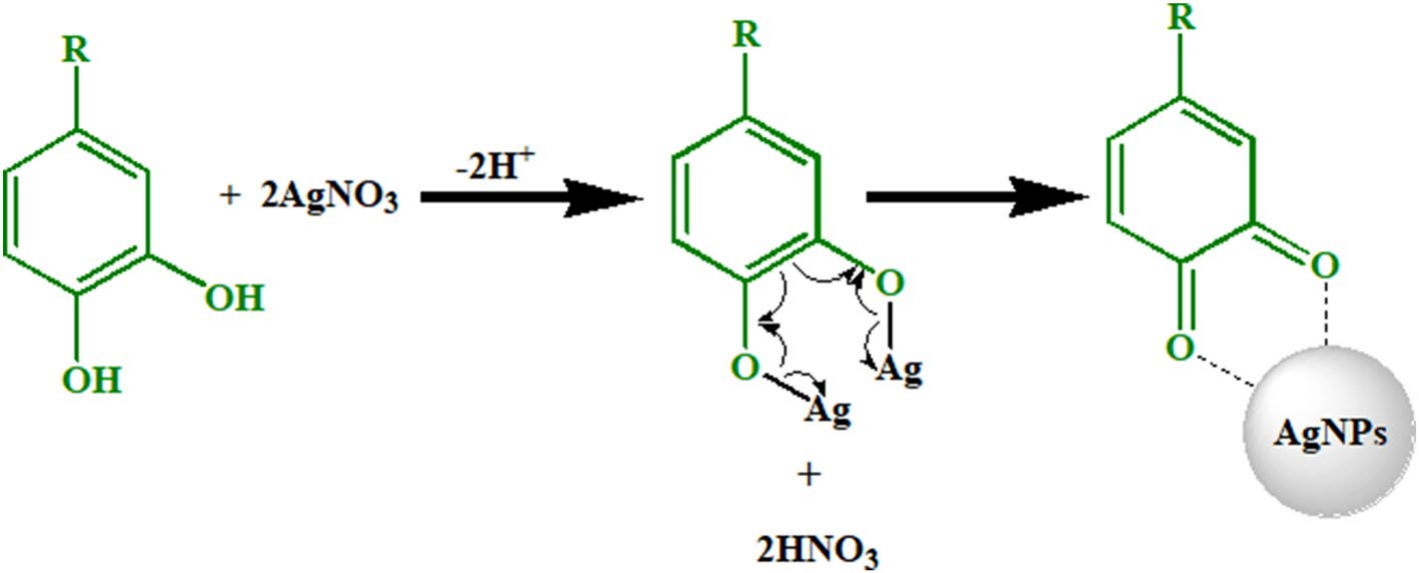
Suggested reduction mechanism of Ag^+^ to Ag^0^.

The plant extract contains various molecules such as polyphenols, terpene derivatives, saccharides, alkaloids, etc. These molecules are responsible for the reduction of AgNO_3_ to Ag^0^. The probable mechanism of AgNPs synthesis is depicted in Fig. 8. Generally, the functional groups such as hydroxyl (–OH) of these biomolecules interact with AgNO_3_. When AgNO_3_ dissolves in water, it dissociates into two ions, Ag^+^ and NO_3_^−^. The acidic nature of OH groups of phytochemicals resulted in donation of H^+^ ions and acquisition of a negative charge. The negative functional groups like O^−^ of phenols interact electrostatically with Ag^+^. This process leads to the reduction of Ag^+^ ions. The NO_3_^−^ ions accept H^+^ from phenolic OH resulted in the formation of HNO_3_. Ag remains in a free metallic state (Ag^0^) to form AgNPs^50^.

### Antibacterial activity

**Figure 9:**
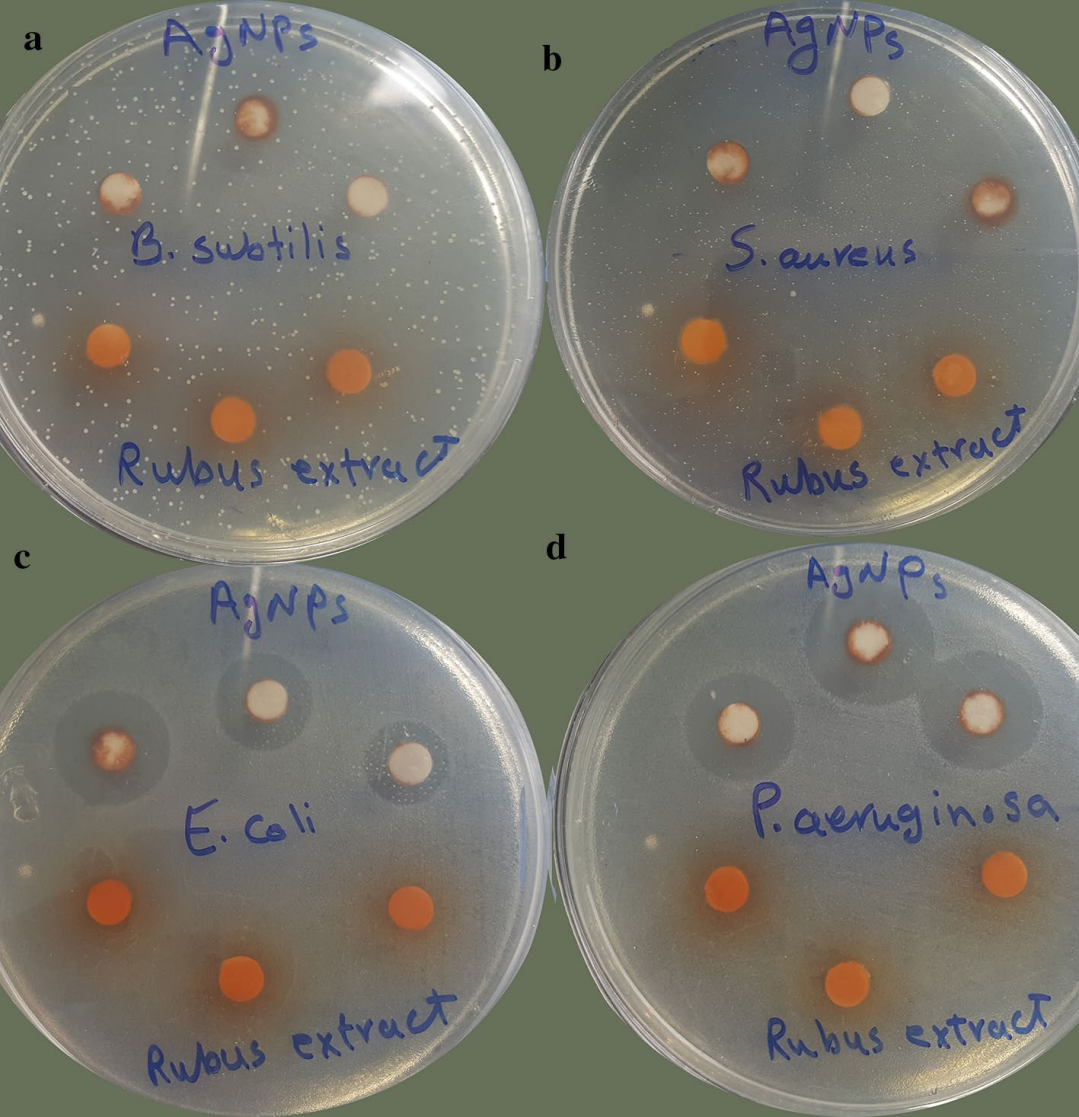
The zones of inhibition formed by the AgNPs and *R. discolor* leaves extract against (**a**) *B. subtilis,* (**b**) *S. aureus*, (**c**) *E. coli*, (d) *P. aeruginosa*.

Green synthesized AgNPs (at concentration of 1 mg ml^−1^) displayed significant antibacterial activity against ATCC gram-negative bacteria, including *P. aeruginosa* and *E. coli*, with inhibition zone of 18 and 16.5 mm, respectively. However, the AgNPs did not show any antibacterial activity against gram-positive pathogens (Fig. 9). The aqueous extract of *R. discolor* exhibited no antibacterial effect even in a high concentration (300 mg ml^−1^). Also, the best MIC value was for *P. aeruginosa* ATCC (0.83 mg ml^−1^) (Table 6).

**Table 6:** Antimicrobial activities of the AgNPs and *R. discolor* leaves extract against ATCC bacteria strain.

Bacteria strain	Zone of inhibition		MIC		MBC	
	AgNPs	extract	AgNPs	extract	AgNPs	extract
*Staphylococcus aureus* (ATCC 6538)	12	–^a^	–	–	–	–
*Bacillus subtilis* (ATCC 9634)	13	–	–	–	–	–
*Pseudomonas aeruginosa* (ATCC 9027)	18 ± 0.9	–	0.83 ± 0.2	–	1.6	–
*Escherichia coli* (ATCC 8739)	16.5 ± 1.2	–	1.6 ± 0.11	–	1.6	–

*ZI* zone of inhibition (mm); *MIC* minimum inhibitory concentration (mg ml^−1^); *MBC* minimum bactericidal concentration (mg ml^−1^); ^a^–: no antibacterial activity reported; the results are the mean ± SD.

Also, the antibacterial activity was measured against MDR *E. coli* and *P. aeruginosa* isolated. Results of the antibiogram susceptibility test of eight antibiotics against ten isolates are depicted in Table 7.

**Table 7:** The antibiogram test of eight antibiotics against MDR *E. coli* and *P. aeruginosa* isolates.

Isolate	PTZ ^(1)^	SXT ^(2)^	FEP ^(3)^	AN ^(4)^	CAZ ^(5)^	MEN ^(6)^	CP ^(7)^	CRO ^(8)^	GM ^(9)^	FM ^(10)^	AM ^(11)^
E1	R	R	I	S	R	R	I	R	S	R	R
E2	R	R	R	R	R	R	R	R	R	R	R
E3	R	R	R	R	R	R	R	R	R	S	R
E4	I	R	S	S	R	R	I	R	R	S	R
E5	R	R	R	S	R	R	R	R	R	S	R
P1	R	R	S	S	R	R	I	R	S	R	R
P2	R	R	R	S	R	R	R	R	R	R	R
P3	R	R	R	S	R	R	R	R	S	R	R
P4	R	R	I	S	R	R	R	R	I	R	R
P5	R	R	R	R	R	R	R	R	R	R	R

*PTZ* piperacillin/tazobactam, *SXT* cotrimoxazol, *FEP* cefepime, *AN* amikacin, *CAZ* ceftazidime, *MEN* meropenem, *CP* ciprofloxacin, *CRO* ceftriaxone, *GM* gentamicin, *FM* nitrofurantion, *AM* ampicillin, *E* *E. coli*, *P* *P. aeruginosa,* *S* susceptible, *I* intermediate, *R* resistant.

**Figure 10:**
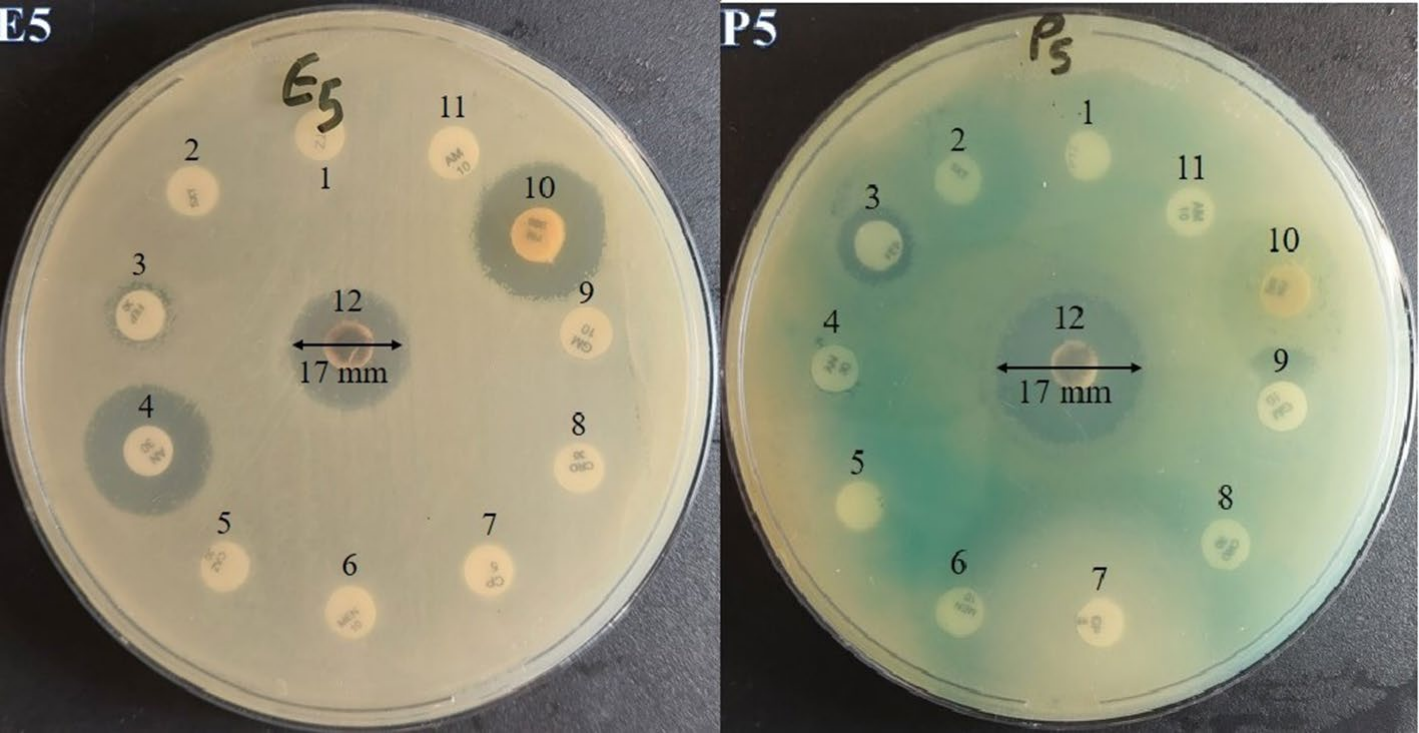
The zone of inhibition (mm) for eleven antibiotics (No. 1–11) and AgNPs (No. 12) against isolate number 5 of MRD *E. coli* and *P. aeruginosa* (E5 and P5).

Also, Fig. 10 describes the zone of inhibition (mm) for eleven antibiotics and AgNPs against isolate number 5 of MRD *E. coli* and *P. aeruginosa*. As is presented in Table 8, the AgNPs showed antibacterial activity against MDR *E. coli* with MIC values ranged from 1.87 to 3.75 mg ml^−1^. The MBCs were 5 mg ml^−1^ for all of the MDR *E. coli* isolates*.* Moreover, the MIC values against MDR *P. aeruginosa* isolated ranged from 0.93 to 1.87 mg ml^−1^, and the MBC values were 2.5–5 mg ml^−1^. The aqueous extract did not have any antibacterial activity against tested MDR isolates.

**Table 8:** The Zone of inhibitions, MIC, and MBC values of AgNPs on *E. coli* and *P. aeruginosa* isolates.

Isolates	Zone of inhibition	MIC	MBC
E1	23	1.87	5
E2	14	1.87	5
E3	16	1.87	5
E4	12	1.87	5
E5	17	3.75	5
P1	21	1.87	5
P2	19	0.93	2.5
P3	19	0.93	2.5
P4	19	1.87	5
P5	17	1.87	5

*E* *E. coli*; *P* *P. aeruginosa;* Zone of inhibition (mm); *MIC* minimum inhibitory concentration (mg ml^−1^); *MBC* minimum bactericidal concentration (mg ml^−1^).

**Figure 11:**
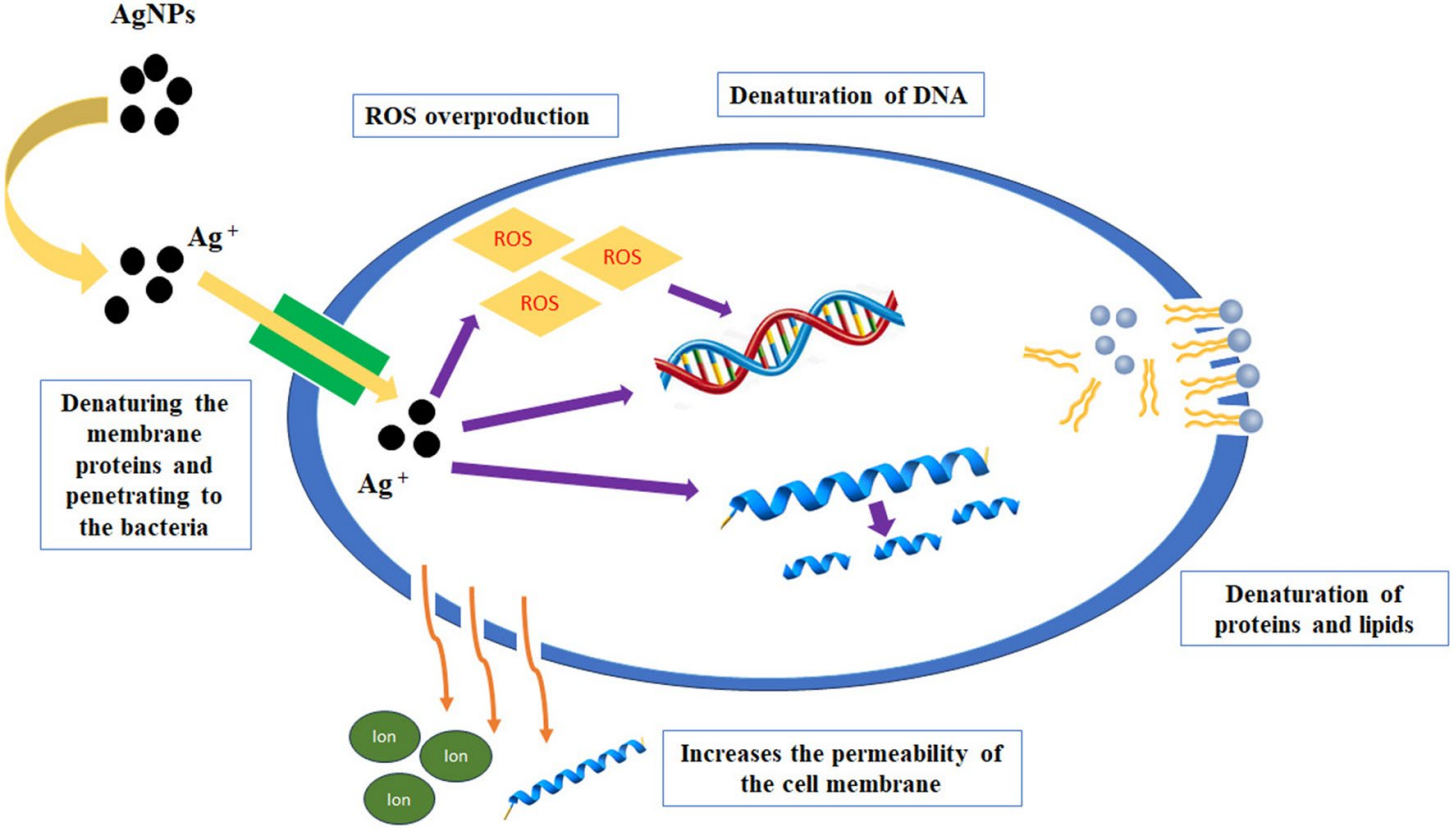
The antibacterial mechanism of action of AgNPs.

AgNPs had ultra-small size and uniform distribution that led to significant antibacterial activity^7^. It is proposed that AgNPs release Ag^+^ that attach to the negative charge of the microbial cell wall, denaturing the membrane proteins. Also, AgNPs have potent affinity for the sulfur-containing proteins in the cell wall, leading to changes in the morphological structure of the cell membrane. This irreversible damage increases the permeability of the cell membrane, thereby disrupting the cell ability to regulate normal activity. This can lead to the loss or leakage of cellular contents such as, proteins, cytoplasm, ions, and cellular energy sources^57^. After crossing the cell membrane, AgNPs disturb the bacteria’s metabolic pathways. They cause several intracellular changes like enzyme inhibition, interaction with bacterial DNA resulting in denaturation of DNA, interruption of the bacteria growth, and inducing electrolyte imbalance^7,15,47^. Another mechanism of action is increasing oxidative stress by inducing overproduction of ROS (reactive oxygen species). ROS can oxidate macromolecules like lipids, DNA, and proteins and therefore, cause the bacterial death (Fig. 11).

Studies have been shown that AgNPs are more effective against gram-negative bacteria strains than gram-positive ones. The suggested reason is that the gram-positive bacteria consist of one cytoplasmic membrane and a relatively thick cell wall that include numerous peptidoglycan layers (thickness between 20 and 80 nm). In contrast, gram-negative strains, there is an external layer of lipopolysaccharide (LPS) as well as one thin layer of peptidoglycan and an internal plasma membrane^58^. Our results are in consistence with previous reports.

### Cytotoxic assay

**Figure 12:**
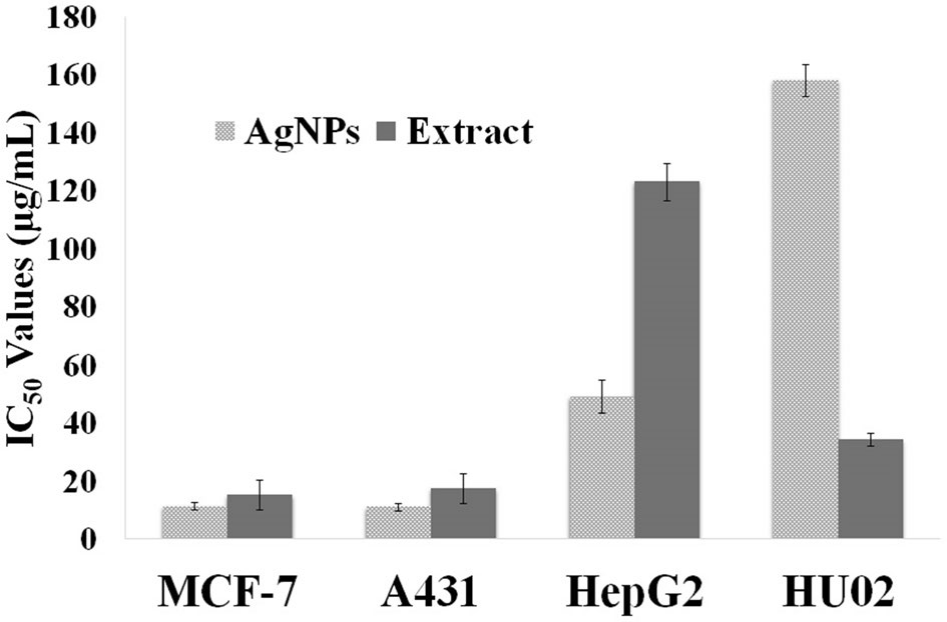
Cytotoxic activities (IC_50_ values) of AgNPs and *R. discolor* leaves extract against MCF-7, A431, HepG2, and HU02; The results are the mean ± SD.

The anti-proliferative effects of silver NPs and the leaves aqueous extract were investigated against three human cancerous cell lines and a healthy cell line. The IC_50_ of AgNPs on selected cancerous cell lines ranged from 11.2 to 49.1 µg ml^−1^ (Fig. 12). The silver NPs exhibited more cytotoxic activities on MCF-7 and A431 cells than on HepG2 cells. Also, AgNPs showed more potent anti-proliferative activity than the aqueous extract on all cancerous cell line, particularly on HepG2 that the cytotoxicity of AgNPs was 2.5 times more than crude extract. Furthermore, the cytotoxic effect of AgNPs was investigated on HU02 (a noncancerous cell line). It was revealed that AgNPs had much less cytotoxic activity against the normal cell line (IC_50_ of 158 µg ml^−1^) in comparison with the extract.

Recently, AgNPs have attracted great attention for their possible use as an anticancer therapeutic agent because of their significant cytotoxic effect on cancerous cell lines, while they are less toxic on normal cell lines^7,38^. It is suggested that the Ag^+^, released from AgNPs, can directly bind to RNA polymerase, disturbing its activity. Another main proposed mechanism of cytotoxicity is the generation of ROS, which leads to intracellular oxidative stress and consequently cell death. It has been observed that the cytotoxicity of AgNPs is size-dependent. The smaller AgNPs can more easily penetrate the cell membrane and interact with different cell parts. Also, it has been reported that the AgNPs, with higher surface area, can sustainably release more concentration of silver cations^38,59^. It has been revealed that the green synthesized AgNPs can carry numerous plant secondary metabolites on their surface that enhance the effectiveness of AgNPs^7,38^. Table 9 summarizes the IC_50_ values of AgNPs prepared from several plant extracts against the same cancerous cell lines as our study. As is presented, the IC_50_ values of AgNPs synthesizes from leaves of *R. discolor* was in the range of previous studies. Also, AgNPs from *R. discolor* had stronger cytotoxic activities against MCF-7, and A431 compared to the AgNPs that are prepared from other mentioned plant extracts in Table 9.

**Table 9:** The comparison of cytotoxic activities of green synthesized AgNPs from leaves of *R. discolor* with other synthesized AgNPs on MCF-7, HepG2, and A431.

AgNPs	Cell line	IC_50_ (μg/ml)	Refs.
*Pisum sativum* (peels)	HepG2	4.0	^46^
*Nigella sativa (*seeds)	HepG2	7.2	^60^
*Rubus discolor* (leaves)	MCF-7, A431, and HepG2	11.2, 11.0, and 49.1, respectively	present study
*Datura inoxia* (flowers)	MCF-7	20.0	^61^
*Mallus domestica*	MCF-7	33.8	^48^
*Annona squamosa* (leaves)	MCF-7	50.0	^62^
*Sambucus ebulus* (leaves)	MCF-7	62.4	^11^
*Trapa natans* (leaves)	A431	64.2	^44^
*Punica granatum* (leaves)	HepG2	70.0	^63^
*Cucurbita maxima* (petals)	A431	82.4	^64^
*Moringa oleifera* (leaves)	A431	83.6	^64^
*Artocarpus integer* (leaves)	MCF-7	90.0	^2^
*Scindapsus officinalis* (fruits)	HepG-2 and MCF-7	155.81, 114.74, respectively	^65^
*Glycyrrhiza uralensis* (roots)	MCF-7	498.93	^66^

## Conclusion

In current study, the biosynthesized AgNPs were characterized using UV–Vis spectroscopy, FT-IR analysis, DLS, TEM, SEM–EDX, and XRD. All these characterizations confirmed the synthesis of AgNPs with average size of 37 nm. The results of the FTIR spectra showed that the phytochemicals present in *R. discolor* extract play a key role in the production of AgNPs. Phytochemical analysis showed that the leaves of *R. discolor* are a good source of phytochemicals, including phenolics, tannins, and flavonoids. Besides, having health-beneficial effects, these compounds have the ability to reduce silver ions, along with surface coating and stabilization of the AgNPs. The study also aimed to optimize the physical parameters and discover the interaction relations between variables affecting AgNPs biosynthesis, using RSM. The experimental results exhibited that all the factors studied were significant for the variable responses. The optimized condition was found to be an AgNO_3_ concentration of 7.11 mM, a time of 17.83 h, a temperature of 56.51 °C, and an extract percentage of 29.22, with a yield of 53.31%. Considering the surge in antibiotic resistance, the AgNPs prepared from *R. discolor* can be potentially used as an antibacterial agent against MDR *E. coli* and *P. aeruginosa* pathogens (MIC 0.93–3.75 mg ml^−1^). The AgNPs depicted significant cytotoxicity against A431, MCF7, and HepG2 (IC_50_ 11.2–49.1 µg ml^−1^), while no significant toxicity against normal cell line was observed. This optimized, low-cost, and environmentally-friendly method is a valuable approach for producing bioactive silver NPs with high yield and small size.

## Material and method

### Materials

Mueller Hinton broth (MHB), Mueller Hinton Agar (MHA), Aluminum trichloride (AlCl_3_), silver nitrate (AgNO_3_), sodium bicarbonate (NaHCO_3_), 3-(4,5-dimethylthiazol-2-yl)-2,5-diphenyl-2*H*-tetrazolium bromide (MTT), and Folin–Ciocalteu’s reagent were obtained from Merk, Germany. Standard compounds, including gallic acid, tannic acid, and quercetin were bought from Sigma Chemical Company (USA). The MCF7, HePG2, and A431 cancerous cell lines were obtained from the Iranian Biological Resource Center (Iran). The normal cell line (Hu02) was purchased from the National Cell Bank of Pasteur Institute (Tehran, Iran). Dulbecco's Modified Eagle Medium (DMEM) was obtained from Gibco. The fetal bovine serum was got from Invitrogen. All other reagents and solvents used were of analytical grade.

### Plant materials and extraction

**Figure 13:**
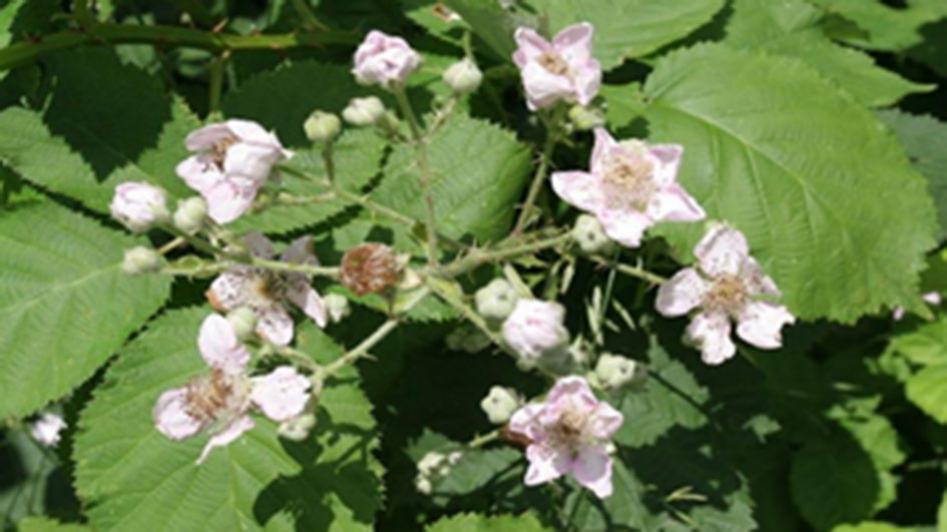
*Rubus discolor* Weihe & Nees.

The aerial parts of *R. discolor* were collected from Fuman-Saravan Road, Guilan province, in the North of Iran, in May 2021 (Fig. 13). The voucher specimen (113 HGUM) was kept in the herbarium of the faculty of pharmacy, Guilan University of Medical Sciences, Rasht, Iran. The plant's leaves were separated from the stems. Then, leaves were shade-dried at room temperature for two weeks and powdered with a mixer grinder. Consequently, 100 g of powder were added to 600 ml of deionized water (DW) and boiled for 15 min. After that, the mixture was cooled and filtered through the Whatman filter paper^7^. Lastly, the solvent was evaporated using a rotary vacuum evaporator (Heidolph, Germany) at 45 °C to obtain 4.1 g of dried extract. It was kept in the refrigerator at 4ºC until required.

### Preliminary phytochemical tests

The preliminary qualitative phytochemical assays were carried out to identify the presence of secondary metabolites in the extract, including flavonoids, tannins, anthraquinones, steroids, carbohydrates, coumarins, and alkaloids, using the standard protocols described by Saeidnia & Ghohari^67^.

### Determination of total phenolic content (TPC)

The Folin-Ciocalteu method was used to measure the total phenolic contents in the extract and AgNPs^68^. In this test, 1 ml of each sample (1 mg ml^−1^) was added to 5 ml of freshly prepared Folin-Ciocalteu reagent (diluted tenfold with distilled water). Then, the mixtures were incubated for 10 min at room temperature before mixing with 4 ml sodium bicarbonate solution (75 g l^−1^). They were incubated for 30 min in the dark. Lastly, the absorbance was obtained at 765 nm using a UV/Vis spectrophotometer. All the experiments were repeated three times. The gallic acid (GA) was used as the reference standard in different concentrations (10, 25, 50, 100, and 150 µg ml^−1^), and the calibration curve was plotted. The total phenolic contents were expressed as mg of gallic acid equivalents (GAE)/g extract.

### Determination of total flavonoid content (TFC)

The measurement of the flavonoid was carried out by the Dowd method^67,68^. First, aluminum trichloride (AlCl_3_) (2%) was prepared in methanol. Then, 5 ml of AlCl_3_ solution was added to 5 ml of each sample (2 mg ml^−1^). The mixtures were incubated for 10 min at room temperature. Finally, the absorbance was measured at 415 nm using a UV/Vis spectrophotometer^7^. All the experiments were repeated three times. The quercetin was used as the standard compound with five known concentrations (10, 25, 50, 75, and 100 µg ml^−1^). Finally, the total flavonoid content was expressed as mg of quercetin as equivalents (QE)/g of extract.

### Determination of total tannin content (TTC)

The aqueous extract and synthesized AgNPs were examined for the total tannin contents by a colorimetric method using polyvinylpolypyrrolidone (PVPP)^69–71^. In this assay, PVPP binds to tannins and precipitates them. Different concentrations of tannic acid (20, 40, 60, 80, 100, 150, and 200 μg ml^−1^) were used for plotting the calibration curve. This method involved two steps. In the first step, 1 ml of each sample (1 mg ml^−1^) was combined with 0.5 ml Folin-Ciocalteu reagent (1 N). Next, sodium carbonate solution (2.5 ml, 20%) was added to each mixture. After 40 min, the absorbance was read at 725 nm. The amounts of total phenols as tannic acid equivalent (X) were calculated using the calibration curve. In the second step, the tannins were removed from tannin-containing samples by adding PVPP (100 mg of PVPP is adequate to bind 2 mg of total phenols). The samples were vigorously shaken (5 min) and kept at 4 °C (15 min). After that, the samples were centrifuged at 4000 g (20 min), and the supernatants were collected. The supernatant only contained simple phenols other than tannins. The phenolic contents of the supernatants were measured, as explained in the first step. The contents of non-tannin phenols (Y) were determined. Lastly, X–Y showed mg of tannin as tannic acid equivalent (TAE)/g extracts.

### Green synthesis of AgNPs

In a typical reaction procedure, different amount of aqueous extract was added to five different concentrations of AgNO_3_ solution (50 ml, 1–10 mM), based on CCD described in the next section. The mixtures were stirred on a magnetic stirrer (Heidolph, Germany) at a constant rate (500 rpm) at different times and temperatures. Next, the mixtures were centrifuged for 15 min at 10,000 rpm using a centrifuge machine^7^. Finally, the sediments were washed three times with deionized water, and dried in a vacuum oven (45 °C). The yield of the AgNPs formation was calculated in optimized condition.

### Experimental design and optimization of AgNPs synthesis by RSM

Previous studies showed that different parameters like concentration of extract and AgNO_3_, time, and temperature have great influence on the size and yield of synthesized AgNPs^72^. In this study, a central composite design (CCD) under Response Surface Methodology (RSM) was employed for the optimization of the most prominent parameters and also for the identification of their cooperative interactions using Design-Expert 7.0 (Stat-Ease, Inc., USA software). Four independent variables were selected, including reaction time (h), reaction temperature (°C), AgNO_3_ concentration, and percentage of extract (%). Each variable was evaluated at five coded levels (− 2, − 1, 0, 1, 2) (Table 10). The total experimental runs were calculated using the following equation: 2^k^ + 2k_+_x_0_, where k is a variable number and x_0_ is the repetition number of experiments at the center point^40^.

**Table 10:** Selected levels of experimental variable in building the CCD.

Parameter	Code	− 2	− 1	0	1	2
Concentration of AgNO_3_ (mM)	A	1	3.25	5.5	7.75	10
Reaction time (h)	B	4	10.5	17	23.5	30
Reaction temperature (°C)	C	30	45	60	75	90
Percentage of extract (%)	D	10	20	30	40	50

### Characterization of AgNPs

The color change from yellowish-green to dark brown was a confirmatory sign for NP formation. Consequently, small sample of the synthesized AgNPs was dispersed in the distilled water and the absorption was measured in the wavelength range of 200–800 nm using a UV–Vis spectrophotometer (PerkinElmer, USA). Distilled water was used as a blank^8^. FT-IR spectroscopy was performed to analyze the surface chemistry and the molecular vibrations of the synthesized AgNPs. The synthesized AgNPs and the extract were screened with a Spectrum Two FT-IR spectrometer with UATR accessory (PerkinElmer, USA) in 400–4000 cm^−1^^6,8^. The TEM was used to determine the morphology (size and shape) of the AgNPs. The microphotographs were obtained using a Zeiss—EM10C—100 kV instrument (Germany). In order to confirm the surface morphology and elemental composition of green-synthesized AgNPs, SEM–EDX instrument was used. A MIRA3 FE-SEM from TESCAN was employed to get SEM images^49^. The preparation of the sample was performed by following and abiding by the manufacturer’s instructions. The size distribution and ζ-potential of NPs was measured by a DLS and Zeta potential analyzer (Nanopartica SZ-100; HORIBA Ltd, Kyoto, Japan). The obtained spectrum provides the hydrodynamic size, distribution, and PDI. For determining the crystalline structure, XRD analysis was carried out using the Bruker AXS model D8 Advance powder X-ray diffractometer ranging from 5° to 80°^7^.

### Antibacterial assay

For investigation of the antibacterial activity of AgNPs and plant extract, two gram-positive (*Staphylococcus aureus* ATCC 6538, *Bacillus subtilis* ATCC 9634) and two gram-negative (*Escherichia coli* ATCC 8739, and *Pseudomonas aeruginosa* ATCC 9027) bacteria were used. In addition, 10 MDR *E. coli* and *P. aeruginosa* isolates, resistant to 8 antibiotics (Gentamicin, Trimethoprim-Sulfamethoxazole, Ceftazidime, Ampicillin, Amikacin, Cefepime, Ceftriaxone, and Ciprofloxacin), obtained from the 17 Shahrivar Children’s Hospital (Rasht, Iran), were used for further investigation of antibacterial properties of AgNPs^73^. The zone of inhibition was measured by the disc diffusion method. Also, minimum inhibitory concentration (MIC) and minimum bactericidal concentration (MBC) were measured by the broth microdilution method using 96 U-shaped well plates^74^.

Bacterial cultures of all bacteria were grown for 24 h in nutrient broth. In agar disc diffusion test, the petri dishes, containing 25 ml of Mueller Hinton Agar (MHA) were used. The agar plates were swabbed with broth cultures standardized with 0.5 McFarland standard solution (1.5 × 10^8^ CFU ml^−1^) of each strain. The sterile discs were located in the agar, and each sample (10 μl) was placed on each disc. The plates were incubated at 37 °C for 48 h. Lastly, the plates were evaluated for the inhibition zones (mm)^75^. All experiments were conducted in triplicate.

For the MIC assay, a stock solution from each sample was prepared in distilled water. Then, a doubling dilution of each sample’s stock solution (100 μl) was prepared in wells using Mueller Hinton broth (MHB). The serial dilutions of samples (extract: 150–0.84 mg ml^−1^, and AgNPs: 10–0.005 mg ml^−1^) were prepared in microplates. Each bacteria inoculum (1.5 × 10^8^ CFU ml^−1^) was diluted in 0.9% saline to give 10^7^ CFU ml^−1^. The plates were spot-inoculated with 100 μl of each prepared bacterial suspension (10^5^ CFU/spot). The bacteria were incubated at 37 °C for 48 h. The plates were tested for the absence or presence of visible growth compared to the negative control wells. The endpoint of MIC was the lowest concentration of the compounds in which there was no visible growth^75,76^. The MBC values were determined by culturing 100 μl of no-growth wells in Petri dishes, contained MHA and incubating at 37 °C for 24 h. The MBCs were reported as the lowest concentration that killed 99.9% of bacterial cells^73^.

### Cytotoxicity assay

Biosynthesized AgNPs and aqueous extract of *R. discolor* were evaluated for anti-proliferative activities at different concentrations in MCF-7, A431, and HU02 cell lines using the MTT assay. Cell lines were cultured in Dulbecco’s Modified Eagles Medium (DMEM). For this reason, 5 × 10^3^ cells per well were seeded in a 96-well plate in complete DMEM and incubated at 37 °C for 24 h in a humidified atmosphere, containing 5% CO_2_. Next, non-adherent cells were removed, and adherent cells were treated with the following concentrations of samples (prepared by serial dilution): 1000, 500, 250, 125, 62.5, 31.2, 15.6, 7.8, and 3.9 μg ml^−1^. The samples were incubated for 48 h. Next, the MTT solution (20 µl, 5 mg ml^−1^ in PBS) and DMEM (180 µl) were added to seeded cells and incubated for 4 h. Then, the supernatants were removed. For dissolving formazan crystals, DMSO (150 µl) was added to each well and shaken for 10 min^75,77^. The optical density (OD) was read at 490 nm using an absorbance microplate reader (BioTek) (the reference wavelength was 630 nm). Each test was repeated three times. The percentage of viable cells was calculated using the following equation:

3 $$ {\text{\% Viability}} = \left( {\frac{{\left[ {{\text{OD treated group}} - {\text{OD background}}} \right]}}{{\left[ {{\text{OD control }}{-}{\text{ OD background}}} \right]}}} \right) \times { }100 $$

IC_50_ of samples (the concentration in which 50% of cells were alive) was calculated using GraphPad Prism (Version 8, GraphPad Software, USA)^77,78^.

## Statistical analysis

Every experiment was carried out in triplicates. All the results are expressed as mean ± standard deviation (SD). The calculation of IC_50_ values (the concentration required for 50% inhibitory activity) was made by nonlinear regression with the normalized fitted dose–response curve (GraphPad Prism Software., version 5, Inc. San Diego, USA)^77^.

### Ethics approval and consent to participate

This study was approved by the Ethical Committee of Guilan University of Medical Sciences (IR.GUMS.REC.1400.093). The collection and use of plant material complies with relevant institutional and national guideline and regulations of plant protection.

## Data Availability

The datasets used and/or analyzed during the current study are available from the corresponding author upon reasonable request.
